# Trastuzumab Deruxtecan for Human Epidermal Growth Factor Receptor 2‐Low Breast Cancer With Cancer‐Related Microangiopathic Hemolytic Anemia: A Case Report

**DOI:** 10.1002/cai2.70029

**Published:** 2025-10-11

**Authors:** Mengyuan Li, Mao Ding, Yukun Hou, Xuening Duan, Yuanjia Cheng, Ling Xu

**Affiliations:** ^1^ Department of Thyroid and Breast Surgery Peking University First Hospital Beijing China

**Keywords:** breast cancer, HER2‐low, microangiopathic hemolytic anemia, trastuzumab deruxtecan

## Abstract

The diagnosis of cancer‐related microangiopathic hemolytic anemia (CR‐MAHA) in patients with breast cancer is often delayed, and this condition is associated with high mortality. A 71‐year‐old female with CR‐MAHA secondary to bone marrow metastasis received trastuzumab deruxtecan (T‐DXd) after first‐line chemotherapy failure and achieved partial hematological remission. Treatment was discontinued because of interstitial lung disease (ILD). Early antitumor therapy is crucial; antibody‐drug conjugates such as T‐DXd offer promise, but vigilance for adverse effects is needed.

## Graphical abstract

The diagnosis and treatment of acquired hemolytic anemia can be challenging. In breast cancer patients, it may be directly associated with the underlying malignancy (as an initial presentation or during disease progression). The diagnosis of cancer‐related microangiopathic hemolytic anemia (CR‐MAHA) is often delayed, and patients have a high mortality rate. A 71‐year‐old female patient was admitted to the hospital due to refractory anemia and thrombocytopenia. After examinations and empirical treatment, a clinical diagnosis of CR‐MAHA secondary to bone marrow metastasis of breast cancer was established. The patient received trastuzumab deruxtecan (T‐DXd) after first‐line chemotherapy failure, achieving partial remission of hematological manifestations, but antitumor therapy was subsequently discontinued due to interstitial lung disease (ILD). Early identification and initiation of appropriate antitumor treatment are the most critical principles in the diagnosis and management of CR‐MAHA patients. Antibody‐drug conjugates (ADCs) offer an alternative to current therapies, but attention to the adverse effects of novel ADCs is equally important.

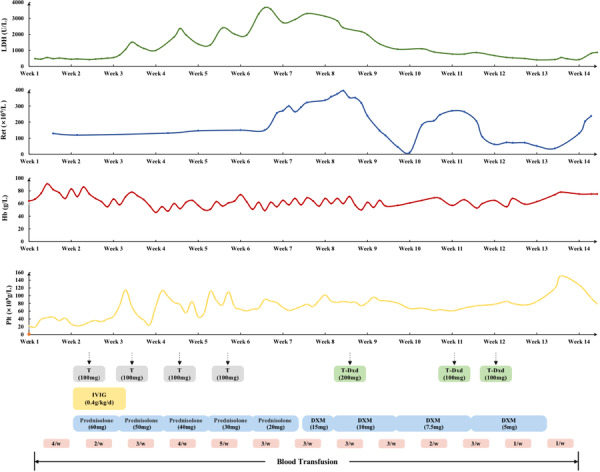

**Abbreviations**
CR‐MAHA: cancer‐associated microangiopathic hemolytic anemia
HER2: human epidermal growth factor receptor 2
HRCT: high‐resolution computed tomography
ILD: interstitial lung disease
TMA: thrombotic microangiopathy

## 1 Background

Thrombotic microangiopathy (TMA) is a group of diseases caused by abnormalities in the arteriole and capillary wall that cause the formation of microvascular thrombi, whose clinical features are microangiopathic hemolytic anemia (MAHA) and thrombocytopenia [1]. While the most common primary forms include thrombotic thrombocytopenic purpura and hemolytic uremic syndrome, cancer‐associated MAHA (CR‐MAHA) represents a rare secondary form associated with malignancies. The pathogenesis of CR‐MAHA remains unclear. Only several case reports [2, 3, 4, 5, 6, 7] and small retrospective cohort studies [8] have reported on this rare paraneoplastic syndrome with a very poor prognosis (Table 1). Timely and effective antitumor treatment may be the only way to improve the outcomes of patients with breast cancer‐associated MAHA, but most patients with end‐stage disease cannot receive cytotoxic drugs because of their poor general condition [8, 11]. However, the development of antibody‐drug conjugates may expand therapeutic options. In this study, we describe a patient with bone marrow metastasis of invasive lobular carcinoma of the breast and MAHA who received T‐DXd after failure of first‐line chemotherapy and achieved partial remission of hematological manifestations.

**Table 1:** Reported cases of MAHA associated with breast cancer.

References	Author	Age	Bone marrow metastasis	Intervention	Prognosis
[9]	Pendse et al.	69	Yes (biopsy confirmed)	Red blood cell and platelet transfusions → hospice care	Died shortly after discharge
[5]	Donato et al.	71	Yes	Supportive care	Died within 24 h of admission
[6]	MGH Case Records	71	Yes (extensive involvement)	Plasma exchange/Rituximab ineffective → Paclitaxel	Transitioned to hospice care, died 1 month later
[4]	Chong et al.	48	Yes (biopsy confirmed)	Plasma exchange + Steroids → Aromatase inhibitor	Outcome unspecified, transferred to oncology for palliative care
[10]	Osti et al.	82	Yes (biopsy positive for metastatic carcinoma)	Steroids + FFP infusion → Palliative care	Clinically stabilized, transferred to hospice care
[8]	Alhenc‐Gelas et al.	57 (median age)	81.5% had bone metastasis	Stratified treatment:	Median OS 28 days
				No antitumor therapy (*n* = 23): Supportive care	No therapy group: Median OS 10 days
				1st‐line antitumor therapy (*n* = 21): Chemo/Endocrine	1st‐line group: Median OS 47 days
				≥ 2nd‐line therapy (*n* = 10): Multiagent chemo	≥ 2nd‐line group: Median OS 290 days

Abbreviations: FFP, freash frozen; MAHA, microangiopathic hemolytic anemia; OS, overall survial.

## 2 Case Presentation

A 71‐year‐old female was hospitalized with refractory anemia and thrombocytopenia without swollen lymph nodes, hepatomegaly, or splenomegaly in January 9, 2023.

### 2.1 History of Breast Cancer

The patient had received modified radical mastectomy for left breast cancer at November 7, 2022 before admission. A routine preoperative examination revealed that her hemoglobin level, platelet count, and kidney function were normal. After surgery, the pathological diagnosis was invasive lobular carcinoma, histological grade III. Immunohistochemical staining confirmed that the tumor was negative for estrogen receptor, progesterone receptor, human epidermal growth factor receptor 2 (HER2) (HER2 1+) and programmed death‐ligand 1 (tumor‐infiltrating immune cells [IC] < 1%), and the Ki67 index was 15%. The comprehensive diagnosis was stage III (pT2N3M0) triple‐negative invasive lobular breast carcinoma, and the patient planned to undergo adjuvant radiotherapy and chemotherapy.

### 2.2 Refractory Anemia With Thrombocytopenia

**Figure 1:**
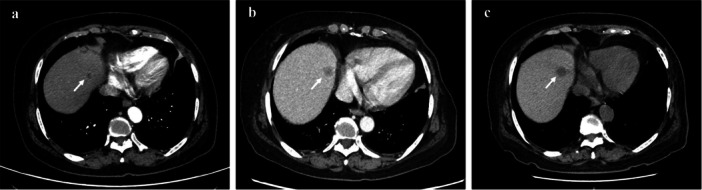
CT scan of the abdomen. (a) CT scan of the abdomen showed a small low‐density nodule with ring enhancement (0.8 cm in diameter) in the S8 segment of the liver. (b) CT scan of the abdomen showed the low‐density nodule in the S8 segment of the liver had increased in size (1.4 cm in diameter). (c) CT scan of the abdomen showed no significant change in the low‐density nodule (1.5 cm in diameter) in the S8 segment of the liver.

**Figure 2:**
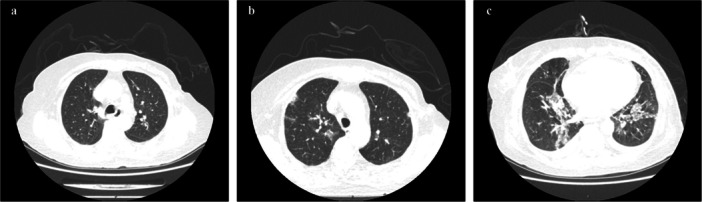
CT scan of the chest. (a) CT scan of the chest showed normal lung. (b) High‐resolution CT of the chest showed multiple ground‐glass density lesions in both lungs after treatment of T‐DXd. (c) High‐resolution CT of the chest showed more and larger ground‐glass density lesions and new sheet‐like consolidation lesions, accompanied by interlobular septal thickening.

**Figure 3:**
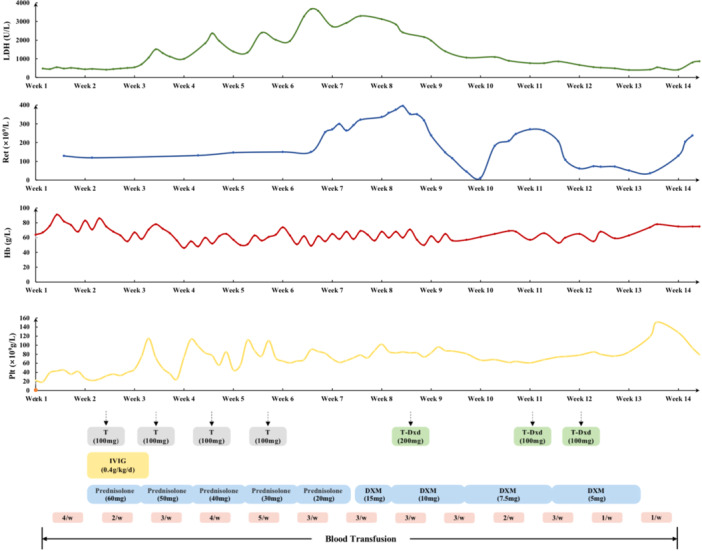
Treatments received and time course of blood tests. DXM, dexamethasone; Hb, hemoglobin; IVIG, intravenous immunoglobulin; LDH, lactate dehydrogenase; Plt, platelet; Ret, reticulocyte; T, albumin‐bound paclitaxel; T‐Dxd, trastuzumab deruxtecan.

**Figure 4:**
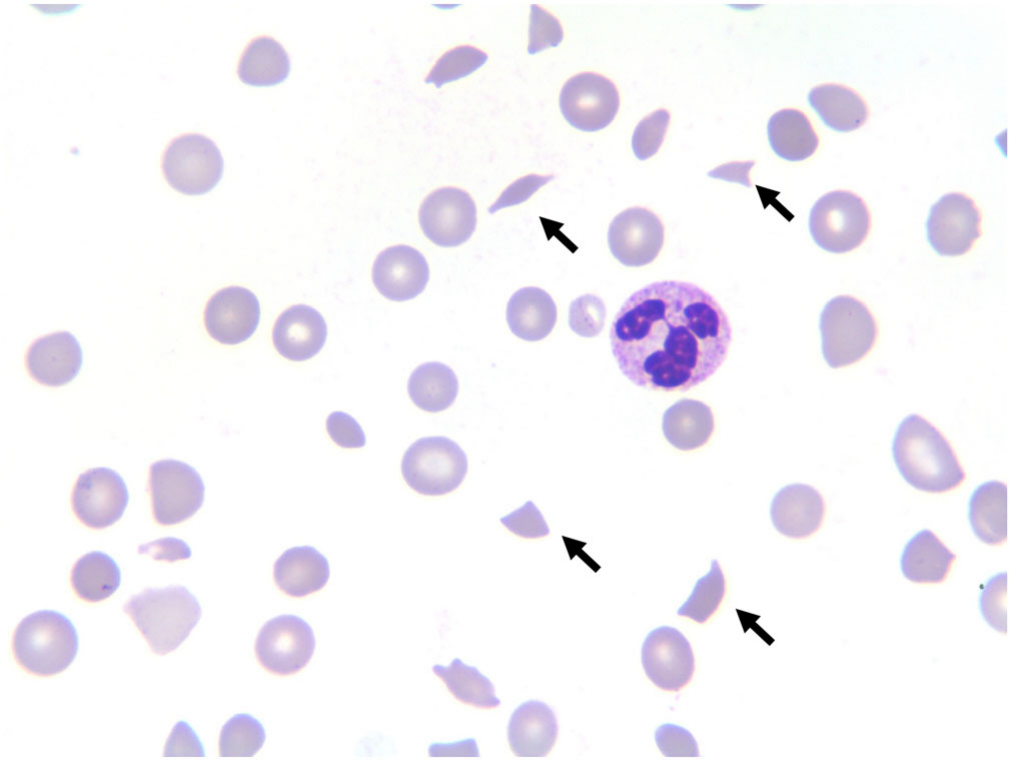
Peripheral blood smear 5 weeks after admission showed heterogeneous sizes of erythrocytes, in addition to a large number of nucleated erythrocytes, schistocytes (> 20%), and polychromatic erythrocytes.

In December 2022, the patient developed severe acute respiratory syndrome coronavirus 2, delaying adjuvant therapy. She experienced syncope and was transferred to another hospital with severe anemia and thrombocytopenia. Dynamic CT revealed a decrease in bone density, a low‐density liver nodule (Figure 1a) and no significant lung abnormalities (Figure 2a), whereas bone marrow biopsy indicated metastasis of the invasive lobular breast carcinoma. Anemia and thrombocytopenia were attributed to bone marrow involvement, leading to the need for multiple blood transfusions (Figure 3). Despite treatment with intravenous immunoglobulin and glucocorticoids, hemolysis persisted, prompting the initiation of nab‐paclitaxel chemotherapy (0.1 g per week). After four cycles, the patient developed dark‐colored urine and an increased need for blood transfusion. A peripheral blood smear revealed schistocytes (Figure 4), but the possibility of thrombocytopenic purpura was ruled out. Glucocorticoid doses were adjusted, yet hemolysis continued, resulting in the gradual discontinuation of glucocorticoids.

### 2.3 Efficacy of First‐ and Second‐Line Treatment

After the diagnosis of CR‐MAHA was confirmed in February 21, 2023, the treatment focus shifted toward antitumor therapy. Unfortunately, nab‐paclitaxel proved ineffective, and increased tumor marker levels at 7 weeks and hepatic lesion progression were observed (Figure 1b). During treatment with nab‐paclitaxel, the patient experienced adverse reactions and could not tolerate further chemotherapy. T‐DXd was chosen as the second‐line salvage therapy. At the 8th week, the initial dosage of T‐DXd was 200 mg. Owing to a urinary tract infection, the second dose was postponed, and the dose was reduced to 100 mg at Week 11. After treatment, laboratory parameters and transfusion requirements decreased (Figure 3), and imaging revealed stable disease (Figure 1c), a decrease in tumor marker levels, and partial alleviation of hemolysis, indicating treatment efficacy.

### 2.4 ILD Complicated With Lung Infection—Discontinuation of Antitumor Treatment

At the 12th week of admission, the patient developed a low fever with cough and sputum production. High‐resolution computed tomography (HRCT) of the chest revealed multiple ground‐glass‐density lesions in both lungs (Figure 2b). Treatment with third‐generation cephalosporins was ineffective. Serial follow‐up chest HRCT showed more severe lung lesions (Figure 2c). Notably, the levels of the fibrosis‐related indicators in the patient's blood were significantly abnormal: hyaluronic acid (1484.16 ng/mL) and type IV collagen (493.01 ng/mL). After consultation of the multidisciplinary team, T‐DXd was stopped because of possible T‐DXd‐associated ILD. The patient refused further examinations and antitumor treatment, entering the best support and hospice care stage.

## 3 Discussion

The mechanisms of bone marrow suppression in breast cancer can be broadly categorized into direct tumor infiltration, paraneoplastic suppression, and treatment‐related effects [8]. Features of bone marrow metastasis in cancer include leukemoid reaction, decrease in two or three lineages of blood cells, and an increased proportion of immature cells (such as reticulocytes, myelocytes, and metamyelocytes) in the peripheral blood. In contrast, CR‐MAHA represents a distinct process in TMA involving microvascular thrombosis, endothelial damage by circulating tumor cells, and excessive von Willebrand factor release [6, 7, 8].

The pathogenesis of CR‐MAHA in HER2‐low invasive lobular carcinoma is multifactorial, with a strong propensity for bone marrow metastasis being a key driving factor [8]. Tumor infiltration disrupts the bone marrow microenvironment, triggering a prothrombotic state through three main processes: endothelial injury, the release of procoagulant factors (e.g., tissue factor) [3, 5], and potential dysregulation of von Willebrand factor multimer dynamics [1, 6]. Despite the frequency of bone marrow involvement in advanced breast cancer, overt CR‐MAHA remains rare (~0.1%), reflecting its unique pathophysiology [8]. Notably, CR‐MAHA may represent the initial clinical presentation of invasive lobular breast carcinoma, a malignancy often associated with frequent and occult bone marrow metastasis. This particular feature makes the diagnosis of CR‐MAHA in this context particularly challenging.

Unlike primary therapies for TMA (e.g., plasma exchange, eculizumab, and glucocorticoids), which are ineffective and potentially harmful in patients with CR‐MAHA, antitumor treatment is essential for improving survival [7, 11]. However, with a nearly 50% mortality rate within 1 month of diagnosis, treatment options after first‐line chemotherapy failure remain poorly defined. In this case, T‐DXd successfully resolved hematological manifestations after nab‐paclitaxel failure. Beyond cytoreduction, T‐DXd exerts its effects through a membrane‐permeable payload enabling a “bystander effect” that targets low‐HER2 tumor cells [7, 8]. By rapidly reducing the tumor burden, it alleviates marrow compression and decreases the levels of procoagulant and inflammatory mediators that drive microvascular thrombosis [3, 5, 11]. This direct targeting of the malignant cause of coagulopathy underscores the importance of timely and effective antitumor therapy, potentially allowing partial recovery of the marrow vascular niche and reduced erythrocyte damage. These findings highlight the critical role of T‐DXd in treating HER2‐low advanced breast cancer accompanied by CR‐MAHA.

However, the adverse effects of novel antibody‐drug conjugates must be carefully considered. In this case, the patient developed respiratory symptoms after T‐DXd treatment, with HRCT findings consistent with ILD. Antitumor treatment was discontinued because of suspected drug‐induced ILD. Importantly, given long‐term steroid and antitumor drug use, opportunistic infections (especially Pneumocystis pneumonia) could not be excluded. According to joint guidelines from the American Thoracic Society and European Respiratory Society, while bronchoalveolar lavage cannot be used to definitively diagnose drug‐induced ILD, it can help exclude other primary causes such as infection and malignancy [12]. Unfortunately, the patient declined bronchoscopy and further intervention.

## 4 Conclusion

In summary, the early identification and initiation of appropriate antitumor treatment are the most important aspects in the diagnosis and treatment of CR‐MAHA. To our knowledge, this is the first case report of HER2‐low breast cancer‐associated MAHA treated using TDxd and thus provides a reference for treatment selection for such cases.

## Author Contributions

**Mengyuan Li:** writing – review and editing (lead), writing – original draft (lead), conceptualization (lead), methodology (lead), data curation (lead). **Mao Ding:** conceptualization (equal), methodology (equal), writing – original draft (equal), writing – review and editing (equal). **Yukun Hou:** investigation (equal), data curation (equal). **Xuening Duan:** investigation (equal), data curation (equal). **Yuanjia Cheng:** investigation (equal), data curation (equal). **Ling Xu:** resources (equal); writing – review and editing (equal).

## Ethics Statement

The study was approved by the Biomedical Research Ethics Committee of Peking University First Hospital (No. 2023 research 210).

## Consent

Written consent for publication was obtained from the patient according to our institutional consent form.

## Conflicts of Interest

The authors declare no conflicts of interest.

## Data Availability

The data that support the findings of this study are available in the supporting material of this article.
